# Unsupervised Medical Entity Recognition and Linking in Chinese Online Medical Text

**DOI:** 10.1155/2018/2548537

**Published:** 2018-04-18

**Authors:** Jing Xu, Liang Gan, Mian Cheng, Quanyuan Wu

**Affiliations:** School of Computer, National University of Defense Technology, Changsha, China

## Abstract

Online medical text is full of references to medical entities (MEs), which are valuable in many applications, including medical knowledge-based (KB) construction, decision support systems, and the treatment of diseases. However, the diverse and ambiguous nature of the surface forms gives rise to a great difficulty for ME identification. Many existing solutions have focused on supervised approaches, which are often task-dependent. In other words, applying them to different kinds of corpora or identifying new entity categories requires major effort in data annotation and feature definition. In this paper, we propose unMERL, an unsupervised framework for recognizing and linking medical entities mentioned in Chinese online medical text. For ME recognition, unMERL first exploits a knowledge-driven approach to extract candidate entities from free text. Then, the categories of the candidate entities are determined using a distributed semantic-based approach. For ME linking, we propose a collaborative inference approach which takes full advantage of heterogenous entity knowledge and unstructured information in KB. Experimental results on real corpora demonstrate significant benefits compared to recent approaches with respect to both ME recognition and linking.

## 1. Introduction

In recent years, due to the rapid development of techniques and the increasing concern of people with their health, many medical websites have emerged which not only provide diverse medical information, including health knowledge and medical news, but also provide the online consultation service about diseases. Some well-known Chinese medical websites are Family-doctor (http://www.familydoctor.com.cn/), Muzhi-doctor (http://muzhi.baidu.com/), Qiuyi (http://www.qiuyi.cn/) and so on, which produce a large amount of medical question and answer (Q&A) data based on real patients and doctors every day. This data contains many real individual cases with high medical value, motivating many medical applications, such as disease prevention and self-treatment.

Medical Q&A data, as unstructured text expression, contains many diverse and ambiguous references to medical entities. The diversity is that an entity is referred to in multiple ways, including aliases and abbreviations. The ambiguity means that different entities have the same surface form. For example, “传染病” (epidemic) could refer to either a disease or a film. This gives rise to a great difficulty in ME identification. Only using entity recognition technology is limited in terms of its ability to effectively mine the data. To fully mine and exploit useful medical knowledge, ME recognition and linking is a good solution. Specifically, it first detects and classifies the ME mentions in text and then understands their meanings by linking the mentions to the correct entities in a given KB. For example, given a text such as “MF, 即骨髓纤维化, 症状为脾肿大⋯” (the symptom of MF, namely, myelofibrosis, is splenomegaly), ME recognition determines that the strings “MF” and “骨髓纤维化” (myelofibrosis) are diseases and that “脾肿大” (splenomegaly) is a symptom. ME linking performs the next step, inferring that “MF” and “骨髓纤维化” (myelofibrosis) actually refer to an entity at URL “http://baike.baidu.com/item/骨髓纤维化” and that “脾肿大” (splenomegaly) refers to an entity at URL “http://baike.baidu.com/item/脾大.”

Medical entity recognition (MER) is a well-known problem which has been studied for decades. Medical entity linking (MEL) is a newer research issue which has attracted much attention because of its importance in many applications, such as understanding medical text, KB construction, and Q&A systems. However, existing works on this topic mainly focus on well-formed English text, such as electronic patient records and medical reports. Few studies have focused on Chinese online medical Q&A text data. The research challenges can be summarized as (1) the online medical Q&A text is characterized by unreliable tokenization, abbreviation, and misspellings. This gives rise to a great difficulty in recognizing the correct entity boundary. (2) It is generally brief, lacking rich context information. This affects the availability of context that can be leveraged to assist the linking. (3) Compared to English, Chinese has more complicated syntax rules, so it is difficult to use solutions for the English language.

In this paper, we design an unsupervised framework that recognizes and links ME mentions in Chinese online medical text, namely, unMERL. To the best of our knowledge, this is the first paper that describes such a comprehensive framework for Chinese medical text. The main contributions of this work are as follows.

1. unMERL utilizes a knowledge-driven approach to detect the ME boundaries, which incorporates the offline and online process, thereby significantly improving recognition performance. In addition, the strategy exploiting the dependency relationships between words can capture the nested and combined medical entities well.
2. unMERL uses an improved classifier based on text feature computation and semantic signature similarity, which can efficiently classify medical entities and further filter nonmedical entities.
3. The linking approach synthetically considers the name similarity, entity popularity, category consistency, context similarity, and semantic correlation between entities, which can better distinguish and determine the candidate entities. In addition, to solve the imperfection problem of the KB, we introduce an incremental evidence mining process, thereby significantly improving the linking performance.
4. We extensively evaluate unMERL for the ME recognition and linking task over real datasets. The experiment results show that unMERL can achieve a significantly higher performance compared to current mainstream methods.

The remainder of this paper is organized as follows.  Section 2 discusses related works; Section 3 presents our framework in detail; Section 4 describes our experiments, results, and discussion; and Section 5 concludes this paper.

## 2. Related Works

Entity recognition has been widely studied in the context of the medical domain. Early works on this topic relied on heuristic rules and lexical resources [1–4]. Based on the name characteristics of medical entities, the researchers encoded and mapped the terms in clinical text to the lexical resources. In particular, the widely applied systems include MedLEE [1], EDGAR [2], and MetaMap [3]. The most well-known medical lexicons included MetaThesaurus [5], MeSH (Medical Subject Heading) [6], and SNOMED-CT (Systematized Nomenclature of Medicine-Clinical Terms) [7]. The Chinese version of SNOMED-CT was published in 1997. The rule-based and lexicon-based systems depended on name regularity and lexicon size, which were restricted to extracting the limited and normative entities. However, by incorporating the dependency relationships between words and the online detection process with a search engine, our approach solves these problems well.

More recently, Zhang and Elhadad proposed an unsupervised approach to biomedical-named entity recognition, leveraging terminologies, syntactic knowledge, and corpus statistics [8]. In addition, the bootstrapping algorithm attracted much attention in the context of medical entity recognition [9, 10]. Bootstrapping is an unsupervised machine learning approach which starts from small sets of seeds or rules and iteratively labels the corpus with them by pattern matching [11]. However, it relies on the quality of seeds and the normalization of the corpus, which easily produces semantic deviation, due to involvement of the incorrect seed categories and irregular context information.

In recent years, many researchers have focused on using statistical machine learning approaches in the medical field. The ME recognition problem is transformed into a sequence annotation or a classification problem. The lexical, syntactic, and semantic features of words are used for training various learning models such as HMM (hidden Markov model) [12, 13], MEM (maximum entropy model) [14, 15], CRF (conditional random field) [13, 16–19], and (structured) SVM (support vector machine) [19]. In addition, to alleviate the limitation of a single model, some researchers proposed the cascading methods [15, 20] which combine multiple models, including CRF, (structured) SVM, and MEM. However, the supervised nature of the machine learning-based approaches relies on a large amount of training corpus which need to be annotated by humans. Besides, it is difficult for the feature set to cover all entity types. As a result, they are usually task-dependent. To solve this problem, we propose an unsupervised approach which leverages syntactic knowledge, corpus statistics, and lexical resources for ME recognition.

Medical entity linking is a newer problem. Some effective approaches to English corpora have been proposed. Glavas exploited semantic textual similarity for linking entity mentions in clinical text [21]. Zheng et al. proposed a collective inference approach which leverages semantic information and structures in ontology to solve the entity linking problem for biomedical literature [22]. Wang et al. proposed a graph-based linking approach which first constructs graphs for mentions, KB, and candidates and then exploits the information entropy and similarity algorithm to link biomedical entities [23]. These approaches are dependent on the context and KB. Therefore, the noise and lack of information in the context reduce the accuracy of the linking. In addition, the graph-based approaches have a high computation cost, and the imperfection of KB also impacts the performance of the linking.

Our linking approach synthetically considers multiple entity knowledge, which is more accurate in distinguishing and determining the candidate entities, with lower computational costs. Moreover, our solution adds the step of extracting the relevant context, to solve the noise problem. To optimize the local KB, we still introduce an incremental evidence mining process with the third KB. Entity linking in the Chinese medical domain has been studied less than entity linking in the English medical domain. To our knowledge, our linking approach is the first solution for Chinese online medical text.

## 3. UnMERL

**Figure 1:**
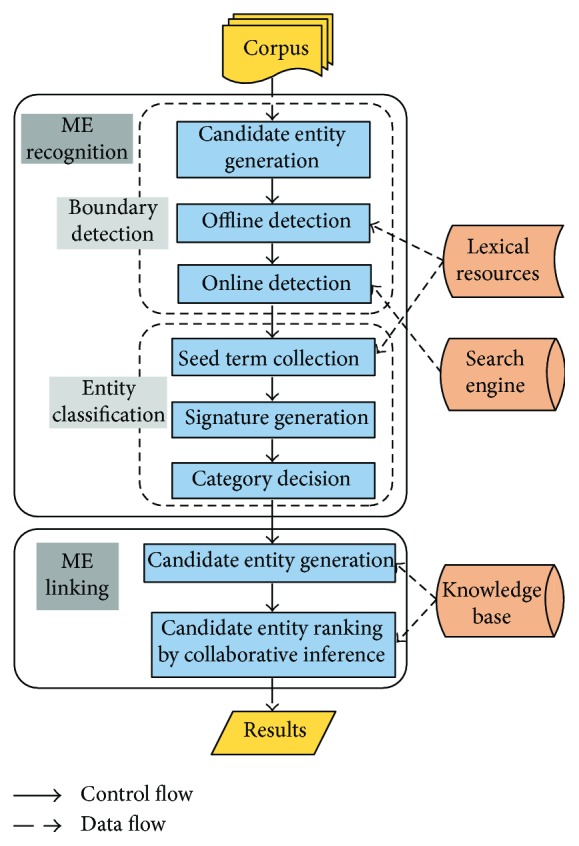
Architecture of unMERL.

The framework of unMERL is shown in Figure 1, in which unMERL consists of two modules. The ME recognition module consumes an input corpus and performs entity boundary detection and entity classification. The output is a set of medical entities and categories. For each recognized medical entity, the ME linking module generates the candidates from the KB and then acquires the target object by ranking them.

### 3.1. Medical Entity Recognition

The ME recognition module aims to detect and classify all ME mentions in the input corpus. Named entity recognition (NER) [24] involves two main steps: detecting entity boundaries and classifying the entities into predefined categories. Based on the thesis, our ME recognition module is implemented in the sequence of two separate processes: boundary detection and entity classification.

#### 3.1.1. Boundary Detection

This step requires the detection of boundaries of medical entities, collecting candidates for entity classification. In our solution, unMERL exploits a knowledge-driven method, mapping the input text to concepts in the lexical resources. Compared to the existing dictionary-based approaches, our approach differs in the following ways: (1) The entity candidates are identified based on the dependency relationships between words. The strategy can well capture the combined and nested entities and reduce the computational cost of the subsequent process by downsizing the candidate set. (2) The search engine is included as a lexical resource, which breaks the conditionality of the limited terms in the dictionary and has good performance in terms of its ability to detect variational and rare entity names. The detection process is roughly divided into two stages: candidate entity generation and medical entity detection.

**Figure 2:**
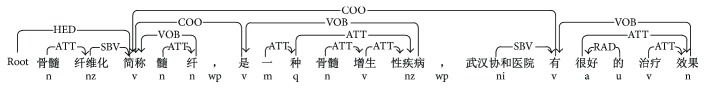
An example sentence with dependency parsing and POS tagging.

*(1) Candidate Entity Generation*. Through corpus analysis, we find that a long medical entity is usually segmented into several fragments by a common nature language processing tool. The POS tag of each fragment is included in Table 1. Moreover, these fragments generally have an attributive dependency relationship. For example, the text “骨髓纤维化简称髓纤, 是一种骨髓增生性疾病, 武汉协和医院有很好的治疗效果” (myelofibrosis, or MF in brief, is a myeloproliferative disease, for which Wuhan Concorde Hospital has a very good therapeutic effect), parsed by the HanLP dependency parser (http://hanlp.linrunsoft.com/), is shown in Figure 2. The dependency labels are shown in Table 1. Based on the hypothesis that entities should be noun phrases (NPs), from the automatically parsed dependency trees, we extract native NPs as candidate entities. A native NP is a single noun (without the attributive modifiers) or a maximum noun phrase with the POS tags in Table 1 and the dependency “ATT.” The candidate entities extracted from the above text are shown in the third row of Table 1.

However, not all noun phrases are medical entities. In order to remove the nonmedical NPs, we employ a knowledge-driven method whose aim is to discover the concepts in the lexical resources referred to in the text. Here, we use Chinese SNOMED-CT [25], the medical KB of Baidu Baike (https://baike.baidu.com/science/medical), and Sogou medical dictionaries (http://pinyin.sogou.com/dict/cate/index/132?rf=dictindex) as the offline lexical resources (LRs). In order to mitigate the limited coverage of the above resources, we still use Baidu Search (https://www.baidu.com/) as an online lexical resource to help recognize the medical entities.

*(2) LR Description*. As mentioned before, Chinese SNOMED-CT, translated from SNOMED-CT (English), is a standard of clinical medicine and contains more than 140,000 clinical terms, covering most aspects of clinical information. To correct incorrect terms in the translated version, we add the medical KB of Baidu Baike and Sogou medical dictionaries. Baidu Baike contains more than 25,000 medical terms edited by authoritative organizations and experts. Sogou medical dictionaries, as the lexicon resource of Sogou's input method, collect data from multiple medical websites. Baidu Search is the largest Chinese search engine, using which we can obtain information that standardized LRs do not cover, such as emerging, rare, and variational medical entities.

*(3) LR Preprocessing*. Considering the heterogeneity and redundancy of the above offline LRs, we extract and fuse the medical terms from them to build a dictionary. In particular, we select specific categories of interest, which are also the goal of our entity classification.  Table 2 presents the statistics in the self-built dictionary. In addition, to improve retrieval efficiency, we build indices by using the first phonetic alphabet of each term.

(1) $$\begin{array}{c} t_{m}=maxOccur\left(r_{1},r_{2},\ldots ,r_{j}\right),\\ r_{j}=\left\{\begin{array}{ll} LCS\left(t_{k},s_{k}\right),\left(k\in K\right),\text{if }Len\left(LCS\left(t_{k},s_{k}\right)\right)>1, & \\ LCS\left(t_{k},t_{i}\right),\left(k,i\in K,k\neq i\right). & \end{array}\right. \end{array}$$

Given a candidate entity, the results returned by Baidu Search contain not only the objective medical term but also other noise information that impacts the performance of the entity recognition. Therefore, we need to process the search results to obtain an unmixed medical term. Based on the common knowledge that there are more correct results than incorrect results, the method is implemented based on corpus statistics and “LCS” (a function of achieving the longest common substring), as shown in (1). Given the search result set *S* = {(*t*_*k*_, *s*_*k*_)}_*k*=1_^*K*^ (*t*_*k*_ represents a title, and *s*_*k*_ represents a summary.), we first get the kernel term from each result by using the “LCS” function. However, not all summaries contain the kernel terms in the titles. Therefore, we add the process LCS(*t*_*k*_, *t*_*i*_) for the search results without common substring. Finally, in the kernel term set, we select the most frequent term *t*_*m*_ as a correct medical term.

In addition, considering that the search engine has no distinguishing ability to filter the nonmedical entities in the candidate set, we establish a medical keyword set. This includes “医” (medicine), “药” (drug), “病” (disease), and “症” (symptom). If a search result contains one or more keywords in the above set, we identify the candidate as a medical entity. If not, it is removed as a nonmedical entity.

*(4) Medical Entity Detection*. Once the medical terms are acquired from the offline and online LRs, detecting the medical entities from the candidate set can be performed. Based on the different characteristics of LRs (that the offline LRs have high accuracy but limited coverage and the online LR have high coverage but lower accuracy), we divide the detection process into offline and online processes. Given the candidate set, unMERL first performs the offline detection with the self-built dictionary. For the output nonmedical candidates, unMERL performs the online detection with Baidu search engine. Here, we exploit the string matching and text distance constraint to implement the detection process.

(2) $$\begin{array}{c} Sim_{r}\left(t_{cm},t_{m}\right)=\frac{Len\left(LCS\left(t_{cm},t_{m}\right)\right)}{\min\left(Len\left(t_{cm}\right),Len\left(t_{m}\right)\right)}, \end{array}$$

(3) $$\begin{array}{c} Dis\left(t_{cm},t_{m}\right)=\left|Loc\left(d_{t_{cm}},t_{cm}\right)-Loc\left(d_{t_{cm}},t_{m}\right)\right|. \end{array}$$

Given a candidate *t*_cm_ and a medical term *t*_m_ in LRs, we use the length proportion of their longest common substring and the shorter term as their name similarity, as (2). The similarity computation can capture the nested entities. For example, for a candidate “胸肌内膜炎” (endomysitis), if *t*_*m*_ is “胸肌” (chest muscle), we can regard “胸肌” (chest muscle) as a nested entity. In addition, considering that some terms and their fractional terms exist together in a text, we use the text distance constraint to improve the detector's accuracy. For example, a text contains both “头孢” (cephalosporin) and “头孢拉定” (cefradine), and the compared medical term is “头孢拉定” (cefradine). For the candidate “头孢” (cephalosporin), if the text distance constraint is not used, the output medical entity is “头孢拉定” (cefradine). Obviously, this is the incorrect surface form for the candidate “头孢” (cephalosporin). In (3), the sign *d*_*t*_cm__ represents the text containing the candidate entity. Function “Loc” computes the location of the second parameter in the first parameter. Using the above two equations, the specific detection process is as Algorithm 1.

In Algorithm 1, the input includes the candidate entity set, the medical term set from the offline and online LRs, and the input text. The output is a set of medical entities. Given a candidate, we first compute its name similarity with each medical term in *MT*. If they are the same, the candidate is regarded as a medical entity. If not, we select the medical terms exceeding the predetermined similarity threshold *θ* for performing the text distance calculation. For each medical term ranked by name similarity, if the text distance between the medical term and the candidate is under the threshold *δ*, the medical term is output as the correct expression of this candidate. In addition, considering the existing of misspelled ME names, we add the “Diff” function to recognize them. This involves counting the number of different characters between a candidate and the medical term (with the highest name similarity). If the number is less than the threshold ϵ (in our experiments, it is set to the number of half the characters in a medical term in our experiments), we output the medical term as the correct expression of this candidate. For example, for a candidate “头孢拉丁” (cefradine), the compared medical term is “头孢拉定” (cefradine), meeting the above condition. Therefore, we output this medical term instead of the candidate.

#### 3.1.2. Entity Classification

Entity categories are additional information for characterizing the entities mentioned. They are essential ingredients in many medical applications, such as medical dictionaries, medical KBs, and medical service systems. Our classification approach is partly inspired by the use of seed knowledge and context signature similarity in [8]. The difference between our approach and the classification approach in [8] is in the following four ways: (1) In the collection of seed terms, we use the framework information in the terminology instead of the category tags, reducing classification error. Meanwhile, we classify some ME mentions based on text feature computation, thereby avoiding the constraint of dissimilar context and the lack of context. (2) Signature vector computation is refined through word embedding, which can better measure the semantic similarity than the TF-IDF method. (3) The filtering threshold is automatically generated by averaging the signature similarity of seed terms, thereby reducing labor costs and increasing filtering accuracy. (4) The seed set is scaled up continually to improve coverage. The classification is implemented by applying the following three steps: seed term collection, signature generation, and category decision.

*(1) Seed Term Collection*. This step involves collecting seed terms for entity categories, based on which the signature vectors of the categories will be generated in the subsequent step. Here, we utilize Baidu Baike to automatically gather the seed terms. In an in-depth analysis, we find that the medical entities of the same class have similar framework information in Baidu Baike, which is more accurate than only using category tag, in identifying the entity category. Therefore, we design a text feature computation-based seed collection approach. Here, we define *T* = {s, a, d, c} as the set of text features, with a subtitle “s,” the attribute names “a” of the infobox, the directory names “d” of the content, and the category tags “c” in the entry page of Baidu Baike. The approach is implemented as follows. (1) From the self-built dictionary, we randomly select 50 terms from each category, to extract and fuse their text features as the category signature. In particular, we exploit the perfect string matching algorithm to produce unambiguous Baidu Baike entries. (2) For each candidate, we also crawl the feature information from Baidu Baike. Then, we calculate its string similarity with all category signatures by using (4) and classifying this candidate to the category with the highest similarity. In particular, the signs *W*_*c*_ and *W*_*cm*_ represent the word sets of a category signature and the feature information for a candidate, respectively. Finally, the classified candidate entities are used as the seeds for the category signature computation in the next step.

(4) $$\begin{array}{c} Sim_{c}\left(W_{cm},W_{c}\right)=\frac{\left|W_{cm}\cap W_{c}\right|}{\left|W_{cm}\right|}. \end{array}$$

*(2) Signature Generation*. This step involves transforming the medical terms (including candidates and seeds) and categories into signature vectors. Here we use the phrase “term signature” to denote the vector of a ME mention or a seed term. Considering that the internal words have descriptive ability for a term, we use the internal and context words for signature generation. To capture the semantic similarity between words, we exploit a word embedding approach to calculate the vector value of a word. Here we use the Word2Vec model, a distributed representation model, to express the words in text as vectors based on deep learning technology [26]. The training corpus is the input corpus, the description content of all medical terms in Baidu Baike, and the search results of Baidu Search. The final term signature vector is computed by averaging all word vectors, in accordance with (5). In addition, we use the phrase “category signature” to denote the vector of an entity category. This is computed by averaging the signature vectors of all seed terms belonging to the same class, following (5).

*(3) Category Decision*. Once all term signatures and category signatures are generated, the category of each candidate is identified by using Algorithm 2. The symbol description is shown in Table 3. The similarity calculation between vectors adopts a cosine similarity algorithm, following (6). Though Algorithm 2, each candidate exceeding the filtering threshold is assigned to the category with the highest similarity. In addition, the filtering threshold is automatically computed by averaging the signature similarity of seed terms, following (7). In particular, ∣*c*∣ is the number of seed terms belonging to a class, corresponding to ∣*t*_*k*_∣ in Algorithm 2. *C*_∣*c*∣_^2^ is the combination function, counting the number of combinations of any two seeds. Finally, to increase the coverage of the seed set, we add the classified candidate to the relevant seed signature set and then update the filtering threshold and the category signature.

(5) $$\begin{array}{c} v^{c}=\frac{1}{\left|S\right|}\sum_{m\in S}v^{m}, \end{array}$$

(6) $$\begin{array}{c} Sim_{\cos}\left(v^{a},v^{b}\right)=\frac{\sum_{i=1}^{I}\left(v_{i}^{a}\times v_{i}^{b}\right)}{\sqrt{\sum_{i=1}^{I}{\left(v_{i}^{a}\right)}^{2}}\times \sqrt{\sum_{i=1}^{I}{\left(v_{i}^{b}\right)}^{2}}}, \end{array}$$

(7) $$\begin{array}{c} F\left(v_{i},v_{j}\right)=\frac{1}{C_{\left|c\right|}^{2}}\overset{\left|c\right|}{\underset{i,j=1,i\neq j}{\sum }}Sim_{\cos}\left(v_{i},v_{j}\right). \end{array}$$

### 3.2. Medical Entity Linking

We use the medical KB of Baidu Baike as a basic KB. To increase the accuracy of the similarity calculation, we use the medical KB of Hudong Baike (http://www.baike.com/sitecategory-10.html) to expand the description information of the entities in this basic KB. The method is as follows: for each entity in KB, we acquire its page from Hudong Baike and then extract the description content and category information.

In accordance with the procedure of entity linking [27], the ME linking module has two stages: candidate entity generation and ranking. For each ME mention, the module first obtains its candidate entities from the KB, and then selects the top candidate (after ranking) as the linking entity. The mentions without linking entities are regarded as NIL.

#### 3.2.1. Candidate Entity Generation

In this stage, our goal is to increase the probability of the candidate set containing a target entity and to control its size. To accomplish the first goal, we use the fuzzy string matching algorithm to compute the name similarity between a mention and all entities in the KB, in accordance with (8). The function “MCC” acquires the most common characters between two strings in order. It can well process the abbreviations and acronyms besides the standard names. The entities exceeding the similarity threshold *α* are included in the candidate set. However, this algorithm may result in a large candidate set.

To reduce the computational cost in the subsequent processing, we introduce the condition of category consistency to control the size. The specific method is as follows: for each candidate, we acquire its text features in Baidu Baike and then compute the similarity between the category signatures acquired in the section of seed term collection, following (4). The candidates under a predefined threshold *β* are removed from the candidate set. This strategy can still well process the terms that have the same name but different meanings. For example, for a ME mention “传染病” (epidemic), its candidate set includes “传染病 (疾病)” (epidemic [disease]), “传染病 (游戏)” (contagion [game]), and “传染病 (电影)” (contagion [film]). Through category constraint, the latter two candidates are removed.

(8) $$\begin{array}{c} Sim_{l}\left(t_{me},t_{e}\right)=\frac{MCC\left(t_{me},t_{e}\right)}{\min\left(Len\left(t_{me}\right),Len\left(t_{e}\right)\right)}. \end{array}$$

#### 3.2.2. Candidate Entity Ranking

This stage aims to acquire the linking entity in the candidate set by ranking using a confidence score. We propose a collaborative inference method which synthetically exploits the name similarity, entity popularity, context similarity, and the semantic correlation between entities.

Specifically, the name similarity of the mention and its candidates is computed using (8). In addition, based on the common knowledge that the most important entity is the most frequently mentioned, we introduce the entity popularity for distinguishing and discriminating between the candidate entities. Here, we utilize the number of visits in the Baidu Baike page to indicate the entity popularity, which is a positive integer (e.g., 15,348). Considering that the entity popularity is not the only decisive criterion, we establish a conversion to ensure its effectiveness and to avoid impacting other measuring conditions. Given the visiting number *n*, the entity popularity is computed as

(9) $$\begin{array}{c} p\left(n\right)=\frac{\left(\left|n\right|\times 10^{\left(\left|n\right|\right)}+n\right)}{10^{\left(\left|n\right|+1\right)}}, \end{array}$$

in which ∣*n*∣ expresses the digit number. For instance, the above integer is translated into 0.515348.

The existing context similarity-based approaches generally extract the words in a fixed window, which ignores the noise information in the context. To increase the description ability of the context words of a mention, we explore a relevant information extraction approach based on the dependency relationships between words. Specifically, this extracts all words that have a dependency relationship with a mention as the context information. For example, in Figure 2, the relevant information of “骨髓纤维化” (myelofibrosis) is “髓纤” (MF) and “骨髓增生性疾病” (myeloproliferative disease). Then, we compute its string similarity with the description content of each candidate by using (4). In particular, the signs *W*_cm_ and *W*_*c*_ represent the context word sets of a mention and a candidate. Of note, before similarity computation, we need to remove the stop words in the context and the description content.

However, the context information acquired by the above extraction approach is limited. It may result in the same context similarity between different candidates. Moreover, some mentions may have no context information. For the mentions, we add the semantic correlation knowledge for ranking based on the hypothesis that the linking entities of the cooccurring entities in text are also correlated, and they have overlapping context information. The special method is as follows: (1) In the context of a mention, we select some ME mentions (with the linking entities) as the collaborators. (2) We extract the anchors and other noun phrases (which are more descriptive than other words) from the description content of these linking entities and the candidates of the mention, respectively. (3) The context similarity between each candidate and all linking entities is computed, and the candidate with the highest similarity is regarded as the target entity.

In conclusion, the confidence score of the candidate entities can be computed by using (10). *λ* is a control factor (the value is 1 or 0), controlling whether the semantic correlation is computed. If the context similarity of each candidate is 0 or the same, *λ* = 1. If not, *λ* = 0. Given a mention *t*_me_ and a candidate *t*_ce_, the linking entity set of the collaborators *L*, the confidence score is computed using

(10) $$\begin{array}{c} CS\left(t_{me},t_{ce},L\right)=Sim_{l}\left(t_{me},t_{ce}\right)+P\left(t_{ce}\right)+Sim_{c}\left(I\left(t_{me}\right),D\left(t_{ce}\right)\right)+\lambda Sim_{c}\left(A\left(t_{ce}\right),\sum_{t_{le_{k}}\in L}A\left(t_{le_{k}}\right)\right). \end{array}$$

The signs “*P*,” “*I*,” “*D*,” and “*A*” express the entity popularity, the relevant context information, the description content, and the special content containing only anchors and noun phrases in the KB, respectively.

**Figure 3:**
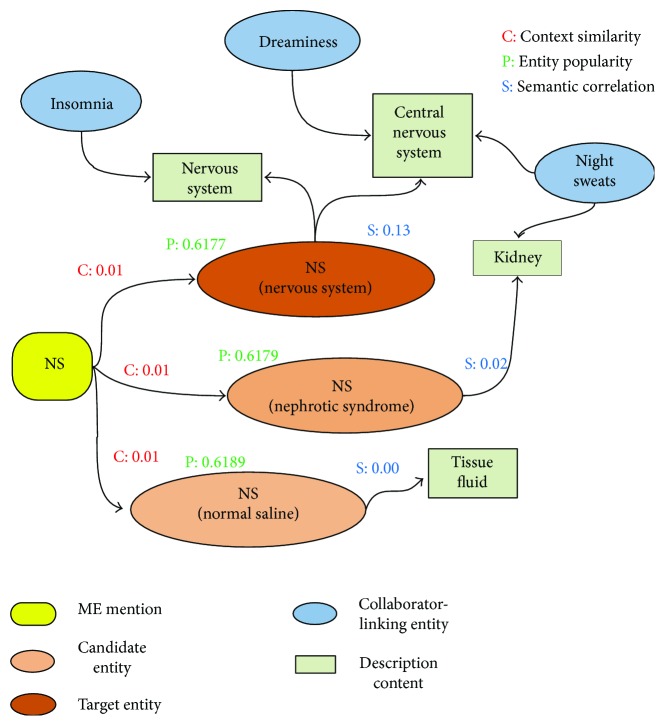
Example of linking the ME mention “NS.”

In order to better understand the ranking process, we provide an example. Given the text “NS,…,功能紊乱体现在失眠, 多梦, 盗汗⋯” (NS,⋯, the dysfunction is reflected in insomnia, dreaminess, and night sweats⋯), the recognized ME mentions are “NS,” “失眠” (insomnia), “多梦” (dreaminess), and “盗汗” (night sweats). Through the previous process, we find that “NS” has multiple candidate entities with the same name, such as “NS (nervous system),” “NS (nephrotic syndrome),” and “NS (normal saline).” Their name similarity is 1, and their other measuring scores are as in Figure 3. It must be noted that we only present the partial value of the entity popularity for the purpose of saving space. According to the confidence scores computed using (10), the candidate “NS (nervous system)” is selected as the linking entity with the highest score (0.7577).

## 4. Experiments

### 4.1. Experimental Data

We crawl 5000 medical Q&A text records from three Chinese medical websites to evaluate our proposed framework, including “家庭医生在线” (Family-doctor), “拇指医生” (Muzhi-doctor), and “求医网” (Qiuyi). Next, we randomly select 500 records from each corpus to recognize all medical entities, classify them to the six categories in Table 3, and link them to the KB manually. In total, we recognize 6596 ME mentions and link 3821 mentions to the correct entries in the KB, whose statistics are shown in Table 4. The sign “NIL” expresses the ME mentions without the linking entities in the KB.

### 4.2. Experimental Evaluation

#### 4.2.1. Comparative Methods

To thoroughly validate the effectiveness of unMERL, we conduct a comparison between the representative state-of-the-art methods and our proposed methods in the recognition and linking modules, respectively. For ME recognition, we select BM-NER [8] and bubble-bootstrapping [10], which are unsupervised methods, as well as Dic-CRF (to distinguish the method, we have given it this name, as it is a supervised method) [18] as the comparative methods. In particular, for the BM-NER method, we use the Stanford parser (https://nlp.stanford.edu/software/lex-parser.html) for chunking. The seed terms are taken from our built dictionary. For the Dic-CRF method, we split 500 records into two subsets: two-thirds for training and one-third for testing. In ME linking, we select QCV (a language independent and unsupervised method) [13] as a comparative method. In addition, it is necessary to state that we use the same seeds in [10] for the bubble-bootstrapping method, the same features in [18] and our built dictionary for the Dic-CRF method, as well as the anchors in the KB to build a KB graph for the QCV method.

#### 4.2.2. Measuring Methods

We use *P* (precision), *R* (recall), and *F*_1_ to measure performance. *P* is the fraction of the correct objects in all objects acquired by the method. *R* is the fraction of the correct objects acquired by the method, in the valid objects in the corpus. *F*_1_ is defined as 2 × *P* × *R*/(*P* + *R*). In addition, we still use “accuracy” to measure the whole linking accuracy, as shown in (11). In particular, ∣*S*_link_∣ and ∣*S*_NIL_∣ express the number of ME mentions that are linked or not linked to the correct entities in the KB by the method. ∣*T*∣ represents the number of ME mentions in the corpus.

(11) $$\begin{array}{c} Accuracy=\frac{\;|\;S_{link}\;|\;+\;|\;S_{NIL}\;|\;}{\;|\;T\;|\;}\times 100\%. \end{array}$$

### 4.3. Experimental Results and Discussion

To simulate ME recognition and linking tasks in an open environment (note: the experimental data has low coverage for real data), we randomly select 30 records for learning all the above-mentioned threshold values. In the ME recognition module, the threshold *θ* for name similarity between a candidate and a medical term in LRs is experimentally set as 0.5. The threshold *δ* for the text distance constraint is experimentally set to 3. This means that if the text distance between a medical term and a candidate is lower than 3, the medical term is output instead of the candidate. In the ME linking module, the thresholds *α* and *β* are experimentally set as 0.5 and 0.47, respectively. In particular, the threshold *α* is used to compute the name similarity between a mention and an entity in the KB, and the threshold *β* is used for category consistency.

#### 4.3.1. Medical Entity Recognition

As mentioned above, our ME recognition module is divided into two stages: boundary detection and entity classification. In order to evaluate the effectiveness of our proposed methods fully, we show the experimental results of each stage in detail.

**Figure 4:**
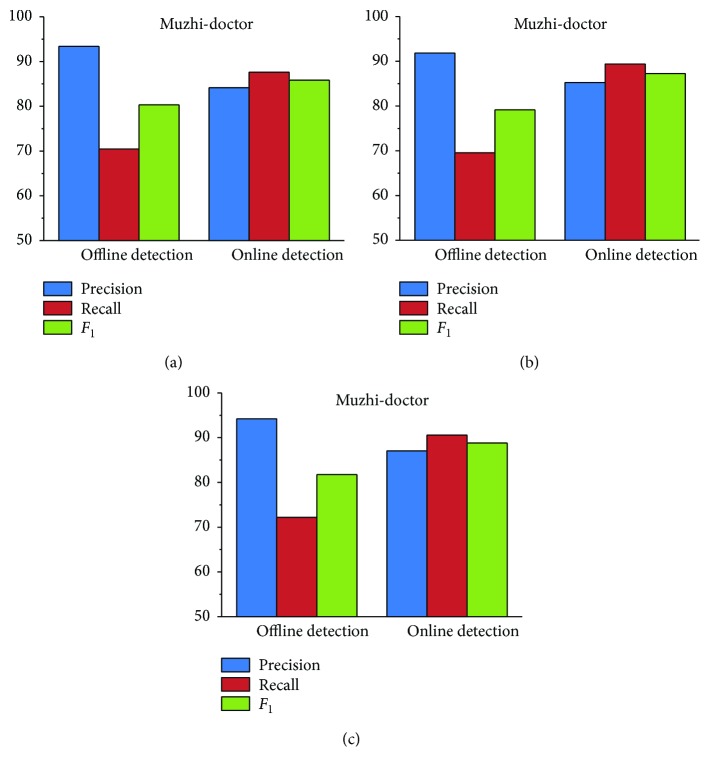
Experimental results after offline detection and online detection on the corpus (%).

*(1) Boundary Detection*. To validate the effectiveness of online detection, Figure 4 presents the experimental results after offline detection and online detection for all datasets. Recall has a noticeable improvement after the online detection process. It is therefore proven that online detection is efficient in solving the limitation problem of the dictionary-based method. However, the precision has some limitations. The main limitation is that some irrelevant terms in the candidate set are not filtered by the online detection process. Therefore, in the entity classification stage, we add the filtering threshold to remove these terms.

*(2) Entity Classification*. To evaluate the entity classification method on its own, we conduct an experiment with the standard entity boundaries for all medical entities in the corpus. Assuming that all medical entities have been extracted correctly from text and that our task is to classify them into the predefined categories, Table 5 presents the classification results of each corpus. The overall performance is significant at an 81.85% precision level and a 75.84% recall level. The lower recall is because when filtering the nonmedical entities, some medical entities are removed by the filtering threshold, thereby reducing the recall. The performance of the target categories “symptom,” “treatment,” and “check” is somewhat low. One possible reason for this is that these entities are mostly classified based on the context signature similarity. However, the lack of the identifying information in the context reduces the similarity score, thus impacting the classification performance.

**Figure 5:**
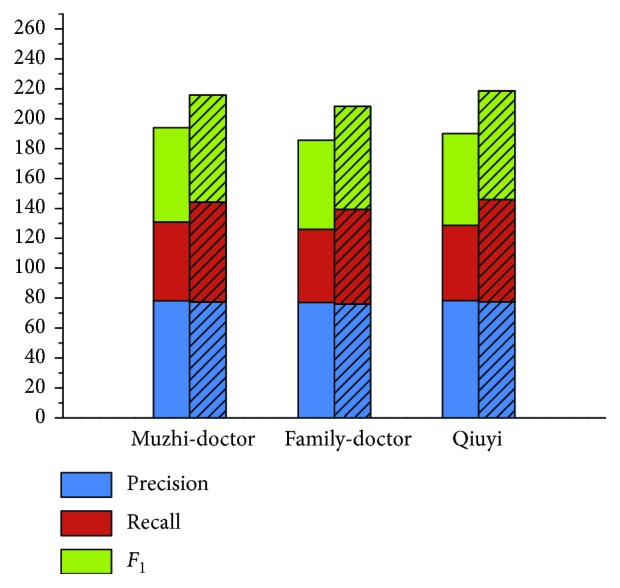
Experimental results of unMER versus bubble-bootstrapping on the symptom category only (note: the cylinder with bias represents unMER, and the other cylinder represents bubble-bootstrapping).

**Figure 6 fig6:**
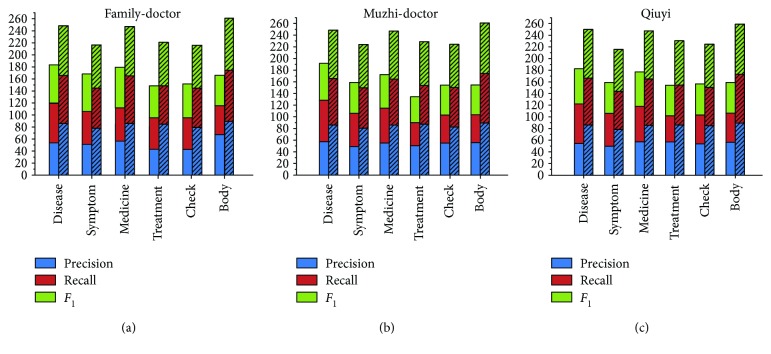
Experimental results of unMER versus BM-NER on the corpus (note: the cylinder with bias represents unMER, and the other cylinder represents BM-NER).

**Figure 7:**
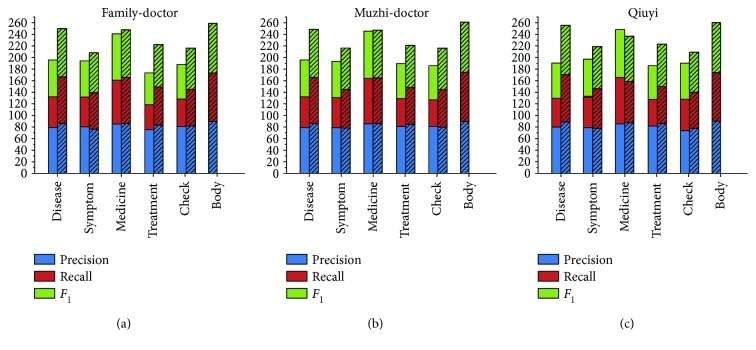
Experimental results of unMER versus Dic-CRF on the corpus (note: the cylinder with bias represents unMER, and the other cylinder represents Dic-CRF).

*(3) Overall Recognition Performance*. We compare the overall performance of our recognition approach (named “unMER”) with the unsupervised and supervised methods described above in Figures 5 [Fig fig6]–7.

Figure 5 shows the experimental results of unMER compared with the bubble-bootstrapping approach. This is because we only acquired the seeds of the symptom category for bubble-bootstrapping. The results show that unMER significantly outperforms bubble-bootstrapping in terms of recall. However, unMER's precision is slightly low. One possible reason for this is that the symptomatic entity mentions are diverse, resulting in low coverage in the offline LRs. Therefore, they are mainly recognized by the online detection method. However, the combined mentions produce diverse search results, from which it is difficult to get a complete term. For example, for the mention “手脚无力” (powerless hands and feet), the returned results contain “手脚无力” (powerless hands and feet), “四肢无力” (powerless limbs), and “手脚发软” (limp hands and feet). After online detection, the acquired entity is “手脚” (hands and feet) or “无力” (powerless). In addition, the low recall of the bubble-bootstrapping approach is because the online Q&A text lacks normalization in its description, reducing the performance of pattern matching.

Figure 6 shows the experimental results of unMER compared with the BM-NER approach. Obviously, unMER outperforms BM-NER in both precision and recall. The value of *F*_1_ of unMER increases 26.12%, 27.52%, and 25.78% on three corpora. The reasons are as follows: (1) The BM-NER approach uses a noun phrase chunker to extract candidate entities, which does not consider the nested entities, thereby reducing the recall. In addition, the chunker utilizes a common NLP tool, which had poor recognition performance for the medical entity boundary. (2) The IDF filter removes many common medical entities. (3) We exploit a distributed word embedding approach to acquire the word vector, which well considers the semantic similarity between words than the TF-IDF algorithm of BM-NER. (4) Our built dictionary contains many incorrect seed categories, and this resulted in semantic deviation for the BM-NER approach, reducing the classification performance.

Figure 7 shows the experimental results of unMER compared with Dic-CRF on each corpus. Note that for the body category, we do not have the features of Dic-CRF and hence do not present its measuring result. On three corpora, the *F*_1_ value of unMER increases 15.01%, 13.68%, and 12.68% than Dic-CRF approach, respectively. By analyzing the experiments, we find that the high recall of unMER mainly depends on the online detection process, which demonstrates the validity of using a search engine for recognizing medical entities. However, Dic-CRF uses a medical dictionary for word segmentation, this can easily lead to incorrect segmentation, especially for the combined entities. In addition, the defined features have low coverage in all entity types, which is also a reason for the low recall. Moreover, the informal description of the online medical text also reduces the recognition performance of the CRF model. In terms of precision, unMER yields comparative results and even exceeds Dic-CRF in some categories. This is due to our combination of multiple offline LRs, thereby increasing the coverage of medical entities. Moreover, unMER has good recognition performance in the nested entities.

#### 4.3.2. Medical Entity Linking

**Figure 8:**
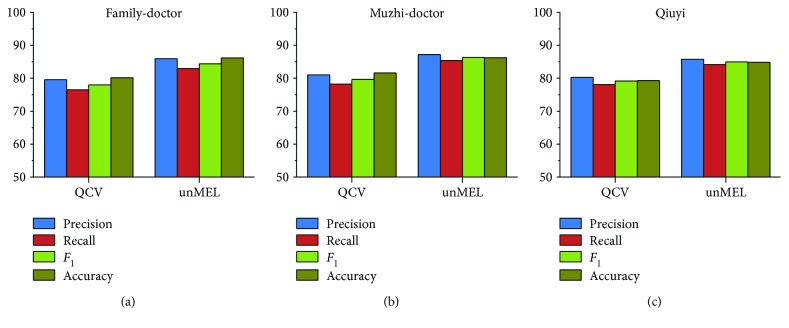
Experimental results of unMEL versus QCV on the corpus (note: the cylinder with bias represents unMEL, and the other cylinder represents QCV).

Figure 8 shows the linking results of our approach (named “unMEL”) compared with the QCV approach on each corpus. To evaluate the linking approach on its own, we conduct an experiment with the standard entity boundaries for all medical entities in the corpus. Assume that all entities have been extracted correctly from text, and our task is only to link them to the correct entities in the KB. Compared to the QCV approach, the *F*_1_ value of unMEL increases 6.39%, 6.67%, and 5.81%, and the accuracy value increases 6.03%, 4.6%, and 5.54% on each corpus, respectively. This is possibly due to the similar relationship in the KB between the mentions within the specific window, QCV virtually uses the context similarity for linking. Therefore, the noise and lack of information in the context reduce the linking performance. However, unMEL alleviates the restriction by extracting the relevant context information and using semantic correlation. Moreover, in the recognition module, we modify the misspelled ME mentions, which help link to the correct entities. Nevertheless, unMEL utilizes the fuzzy string matching to generate candidate entities, which omits some target entities that are fully different in the surface form, reducing the linking recall.

#### 4.3.3. Overall System Performance

To evaluate the overall performance of our framework (unMERL), Table 6 shows the linking results by conducting an experiment with our recognized entities. Compared to the above linking results, both the precision and recall show some decline. The reason is that unMERL obtains some inexact entities in the boundary detection step. In addition, unMERL removes some medical entities when filtering the nonmedical terms in the classification step.

## 5. Conclusions

Medical entity recognition and linking are challenging tasks in Chinese natural language processing. In this paper, we have described an unsupervised framework for recognizing and linking medical entities from Chinese online medical text, namely, unMERL. To the best of our knowledge, this is the first complete unsupervised solution for Chinese medical text with both medical entity recognition and linking. It has considerable value in many applications, such as medical KB construction and expansion, semantic comprehension of medical text, and medical Q&A systems. Experimental evidences show that unMERL consistently outperforms current approaches. In addition, due to its unsupervised nature and language independence, unMERL has good generalizability.

In the future, we will improve unMERL in the following ways. Firstly, we will improve the online detection approach, by adding in-depth textual analysis in extracting medical terms from the search results. Secondly, we will improve the linking approach by introducing semantic analysis.

## Figures and Tables

**Algorithm 1:**
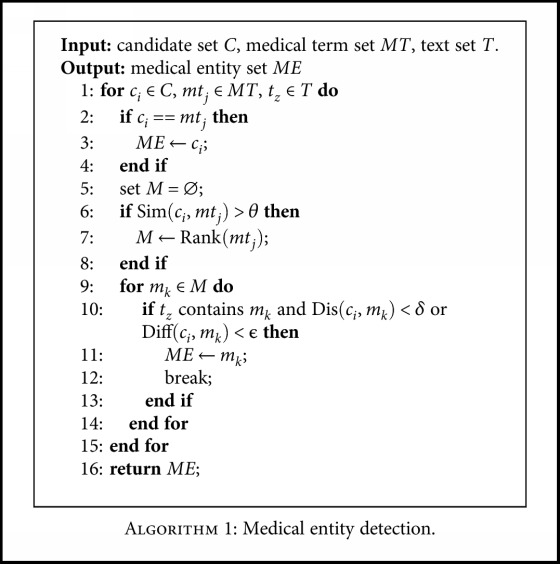
Medical entity detection.

**Algorithm 2:**
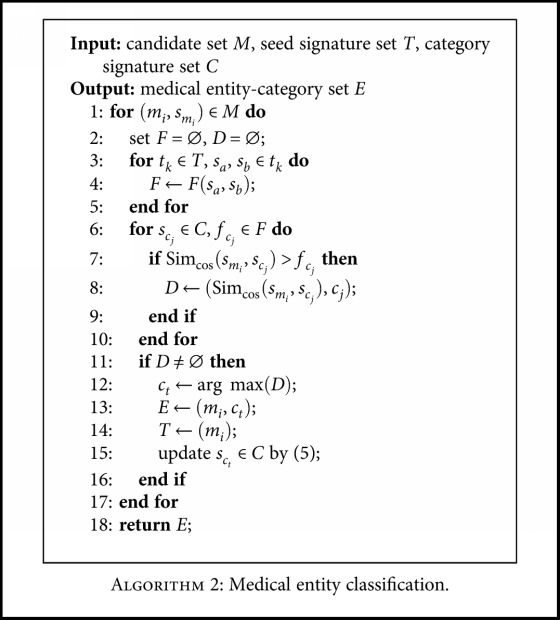
Medical entity classification.

**Table 1:** Constraints on POS tags, description of dependency labels, and candidate entities of the sentence in Figure 2.

Notation	Description
POS tags	f (preposition of locality), m (measure word), b (distinguishing word), rr (personal pronoun), v (verbal word), gb^∗^ (word related to biology), or n^∗^ (noun)
Dependency labels	HED (head), SBV (subject-verb), VOB (verb-object), ATT (attribution), COO (coordination), RAD (right adjunct)
Candidate list	骨髓纤维化 (myelofibrosis), 髓纤 (MF), 骨髓增生性疾病 (myeloproliferative disease), 武汉协和医院 (Wuhan Concorde Hospital), 治疗效果 (therapeutic effect)

**Table 2:** Statistics of the user-defined dictionary.

Category	Term number
Body	1802
Disease	48,120
Symptom	3698
Medicine	42,047
Treatment	7403
Check	768

**Table 3:** Symbol description in Algorithm 2.

Symbol	Description
*M*	A set containing each candidate entity *m*_*i*_ and its signature vector *s*_*m*_*i*__
*F*	A threshold set filtering the nonmedical entities
*t*_*k*_	A seed signature set of the same class
*s*_*a*_, *s*_*b*_	Seed signature
*c*_*j*_, *c*_*t*_	Category name
*s*_*c*_*j*__, *s*_*c*_*t*__	Category signature of *c*_*j*_ or *c*_*t*_
*f*_*c*_*j*__	Filtering threshold of *c*_*j*_

**Table 4:** Statistics of the corpus.

Corpus	MEs	MEs linking to KB	NIL
Family-doctor	2531	1524	1007
Muzhi-doctor	1876	109	780
Qiuyi	2189	1201	988

**Table 5:** Entity classification results on the corpus (%).

Entity category	Family-doctor			Muzhi-doctor			Qiuyi		
	*P*	*R*	*F*_1_	*P*	*R*	*F*_1_	*P*	*R*	*F*_1_
All	82.49	79.13	80.78	80.91	73.86	77.22	82.16	74.35	78.06
Body	85.17	81.20	83.14	83.62	80.13	81.84	83.53	80.21	81.84
Disease	80.45	82.16	81.30	81.96	82.67	82.31	79.26	81.68	80.45
Symptom	78.19	60.84	68.43	74.54	61.73	67.53	76.26	61.52	68.10
Medicine	82.31	79.62	80.94	80.63	75.84	78.16	84.57	78.39	81.36
Treatment	76.86	67.25	71.73	75.24	63.59	68.93	76.61	61.82	68.42
Check	75.14	65.53	70.01	74.50	67.26	70.70	73.48	63.72	68.25

**Table 6:** Experimental results of unMERL on the corpus (%).

Corpus	unMERL			
	*P*	*R*	*F*_1_	*A*
Family-doctor	82.64	73.26	77.67	83.23
Muzhi-doctor	83.37	74.41	78.64	83.48
Qiuyi	82.15	72.79	77.19	82.05
